# 2020: A year of change

**DOI:** 10.1016/j.eclinm.2020.100699

**Published:** 2020-12-17

More than 60 million SARS-COV-2-positive people and more than 1·5 million deaths later, 2020 has been dominated by the COVID-19 pandemic. While social distancing, sickness, and disruption to normal life have affected the work of millions globally, there has been a number of positive events and discoveries made over the year. As we move toward the end of the year, we highlight some of the most important events of 2020.

At the turn of the year news of a wild measles outbreak in a number of south Pacific islands the most prominent of which was Samoa. By Jan 22^nd^, 2020, 5707 measles cases and 83 measles-related deaths had been recorded in the region. The outbreaks were directly related to a decline in levels of vaccination between 2013 and 2018 from an estimated 99% of children younger than 1 year to 40% in 2018 in Samoa. Control of the outbreak was directly addressed via a number of mandatory vaccination programmes across Samoa, Tonga, and Fiji, assisted by an international coalition of nations and organisations including New Zealand, Australia, the UK, French Polynesia, Israel, Hawaii, and UNICEF. The last case of measles in Samoa was reported at the end of January demonstrating the power a strong international collaborative response can have in the control of a lethal viral epidemic.

In March, *EClinicalMedicine* launched a collection of articles highlighting gender disparities and inequality across the world. Following on from *The Lancet*’s theme issue on women in science, medicine, and global health in 2019, this collection took up the commitment to fighting for true parity in gender across the medical and health-care landscapes. The journal continues to be a home to studies and pieces that challenge the entrenched thinking of the health-care landscape.

Inequity and inequality continued to be the theme over the following months, as the murders of Breonna Taylor in March and George Floyd in May brought into focus the continued mistreatment of Black lives across society. The Black Lives Matter movement has been involved in drawing attention to numerous killings and racially motivated violence against Black people since 2013, after the murder of Trayvon Martin. However, the succession of graphic deaths disseminated via social media led to a net-positive response to the movement's advocacy for the importance of Black life and encouraged self-reflection and acknowledgment of the role in racial discrimination across a society. In health care, the self-reflection has triggered researchers to investigate and detail the for too long undiscussed inequities faced by ethnic minorities in health-care systems. These inequalities have been further highlighted by the COVID-19 pandemic and the disproportionate burden on ethnic minorities. *The Lancet* group published a pledge to improve representation and to eliminate systemic racism in health and research from its internal Group for Racial Equality in October. *EClinicalMedicine* aims to play a central role in this commitment by the curation of a collection of articles highlighting ethnic and racial inequity and inequality in healthcare and health-care systems and a call for action and change in 2021.

In August WHO certified the Africa region as free from polio. This certification indicates a 4-year period of polio-free positive cases in the region. The last incidences were reported in Nigeria, which accounted for nearly half of all cases in the continent, in 2010. This eradication milestone represents an enormous vaccination effort across the continent and brings us closer to a polio-free planet.

2020 also revealed progress in the challenges of early testing for neurodegenerative disorders. In October, *EClinicalMedicine* published a study by Elif Eyigoz and colleagues at the IBM Thomas J. Watson Research Center which used artificial intelligence to predict Alzheimer's and cognitive decline using linguistic performance in a standardised task. In November, the above was followed up by the announcement of a blood test available clinically to help diagnose Alzheimer's disease in the USA.

2020 was also the year of the US Presidential election, held in November. As the USA saw the most positive cases and fatalities, much of the election discussion focused on how the COVID-19 pandemic had been handled and future directions. On the eve of the election, *EClinicalMedicine* published an Editorial putting into focus failures in handling the pandemic and calling for a return to science-based decision making. The results of the election and subsequent new health-care appointments suggest a positive change in direction of the US handling of the COVID-19 pandemic moving forward.

November saw Scotland become the first country in the world to provide free and universal access to period products. Recent UK research suggests 15% of girls have struggled to afford sanitary wear, while 14% have had to ask to borrow sanitary wear from a friend due to affordability issues. This simple piece of legislation directly addressed the gender inequity of Period Poverty.

The COVID-19 pandemic galvanised the infectious disease research community to first isolate and identify the virus and at rapid pace construct, test, trial, and manufacture a number of viable vaccines. The unprecedented speed and efficiency shown by research groups and organisations across the globe to develop a vaccine is an incredible example of collaborative science at work. The efforts of these groups on Dec 8, saw Maggie Keenan, a grandmother aged 91 years, receive the first non-trial vaccination as part of the UK's vaccination programme.

For many, 2020 will be a year to forget. In our rush to put this year behind us it is essential we do not forget the sacrifices millions have made. From frontline workers to the millions who have fallen ill or have died from COVID-19, to the further millions who have endured lockdowns, job insecurity, and isolation. We must take a moment to remember these sacrifices and continue to support each other. As the vaccines are distributed, immunity builds and the immediate threat from COVID-19 recedes, we must keep in mind that in an ever-closer connected world we are not truly free of this pandemic until the entire planet is COVID-19 free. There is much to be proud of in the achievements obtained this year and it will be of the utmost importance that the plans put aside in 2020 are carried through with the same determination we have used in enduring this year.***EClinicalMedicine***

